# Laparoscopic counterclockwise modular mesohepatectomy for hepatocellular carcinoma: a standardized anatomical approach (with video)

**DOI:** 10.3389/fonc.2025.1599403

**Published:** 2025-06-18

**Authors:** Xi Chen, Wei He, Jianjun Wang, Ming Kuang, Ting Jiang, Hua Luo, Zhaohui Hu

**Affiliations:** ^1^ Department of Hepatobiliary Surgery, Mianyang Central Hospital, School of Medicine, University of Electronic Science and Technology of China, Mianyang, China; ^2^ Department of Stomatology Mianyang Central Hospital, School of Medicine, University of Electronic Science and Technology of China, Mianyang, China

**Keywords:** hepatocellular carcinoma, laparoscopic liver resection, anatomical liver resection, mesohepatectomy, Laennec’s capsule, surgical technique

## Abstract

**Background:**

Laparoscopic liver resection (LLR) is increasingly utilized for hepatocellular carcinoma (HCC). However, laparoscopic anatomical resection of Couinaud segments 4, 5, and 8 remains technically demanding due to complex vascular anatomy and a broad transection plane.

**Methods:**

This study retrospectively analyzed patients who underwent laparoscopic counterclockwise modular mesohepatectomy (LCMM) at our center. The LCMM approach standardizes the dissection sequence, optimizes vascular control, and utilizes Laennec’s capsule theory to facilitate safe and precise anatomical liver resection. Perioperative outcomes, including operative time, intraoperative blood loss, and postoperative complications, were assessed.

**Results:**

A total of 12 patients with centrally located HCC underwent LCMM. The mean operative time was 253.66 ± 52.47 minutes, and the mean intraoperative blood loss was 177.91 ± 112.76 mL. No conversions to open surgery or intraoperative transfusions were required. The mean postoperative hospital stay was 9.83 ± 4.26 days. Postoperative complications occurred in three patients (one bile leakage, one pulmonary infection, and one posthepatectomy liver failure). No perioperative mortality occurred. The mean disease-free survival (DFS) was 18.75 months.

**Conclusion:**

LCMM appears to be a technically effective and anatomically guided approach for managing centrally located HCC. It facilitates intraoperative control of key vascular structures and yields promising short-term oncological outcomes. Further prospective studies are warranted to confirm its long-term efficacy.

## Introduction

Hepatocellular carcinoma (HCC) is one of the five most common malignancies worldwide and the third leading cause of cancer-related mortality (1). Surgical resection remains the most effective curative treatment for HCC (2). In recent years, the rapid advancement of laparoscopic techniques and improved understanding of hepatic anatomy have led to the widespread adoption of laparoscopic liver resection (LLR) for treating HCC (3, 4). Compared with open liver resection (OLR), LLR is associated with several well-established short-term advantages, including reduced intraoperative blood loss, faster postoperative recovery, and less postoperative pain. Importantly, numerous studies have demonstrated that LLR achieves comparable long-term oncological outcomes to OLR, with no significant difference in overall survival (OS) or disease-free survival (DFS) (5–8). Consequently, LLR is now recommended in clinical practice guidelines for selected patients with HCC (9–11). However, laparoscopic anatomical mesohepatectomy—defined as the resection of Couinaud segments 4, 5, and 8—remains one of the most technically challenging procedures in LLR (12, 13). The difficulty arises from the extensive transection surface, challenges in delineating precise resection margins, and the close proximity to major hepatic vessels, including the middle hepatic vein (MHV) and right hepatic vein (RHV). These anatomical constraints significantly elevate the risk of vascular injury, major intraoperative hemorrhage, and carbon dioxide (CO_2_) gas embolism. As a result, centers with limited experience often report higher conversion rates to open surgery and increased postoperative complication rates (14).

To overcome these technical challenges, we developed a laparoscopic counterclockwise modular mesohepatectomy (LCMM) technique, specifically tailored for centrally located HCC. This method standardizes the procedure by optimizing the dissection sequence and surgical steps, aiming to reduce operative complexity and shorten the learning curve. It fully leverages the inherent advantages of laparoscopy—such as magnified visualization, multi-angle instrument access, and precise manipulation in confined anatomical spaces—to achieve reliable exposure of key vascular structures while minimizing intraoperative complications.

In addition, laparoscopic surgery offers a natural “no-touch” advantage by limiting liver mobilization and direct tumor manipulation, which may reduce the risk of tumor cell dissemination and intrahepatic metastasis compared to open techniques. The LCMM technique therefore represents a structured and anatomically guided approach to minimally invasive liver resection, with promising clinical applicability in experienced centers.

## Materials and methods

### Patients

All consecutive patients diagnosed with hepatocellular carcinoma (HCC) who underwent laparoscopic anatomical mesohepatectomy using the LCMM technique at our hospital between January 2022 and December 2024 were enrolled in this study. Mesohepatectomy was defined as the anatomical resection of liver segments 4, 5, and 8 (15). The indication for anatomical resection was based on the preoperative diagnosis of malignancy or suspected malignant lesions. This study was approved by the Ethics Committee of our hospital (Approval number: S2022099). Written informed consent was obtained from all patients and their families.

All procedures in this series were performed by a single experienced hepatobiliary surgeon specialized in laparoscopic anatomical liver resection, ensuring consistency and minimizing operator-related variability.

### Surgical indications and contraindications

The inclusion criteria for laparoscopic counterclockwise modular mesohepatectomy (LCMM) were as follows: (1) Solitary or multifocal hepatocellular carcinoma (HCC) confined to Couinaud segments 4, 5, and/or 8, in which radical resection could be achieved while preserving an adequate future liver remnant (FLR); (2) Child–Pugh grade A or B liver function; (3) No evidence of distant metastasis on preoperative imaging; (4) No history of prior liver resection or other malignancies; (5) Patient eligibility and informed consent for laparoscopic surgery. Contraindications included: (1) Macroscopic vascular tumor thrombus involving major vessels on imaging; (2) Poor liver function (Child–Pugh > B or ICG-R15 >15%); (3) Insufficient FLR volume (<30%); (4) Presence of extrahepatic metastasis. During the same study period, several patients with centrally located tumors underwent other types of anatomical or non-anatomical liver resections due to not meeting the above criteria (e.g., tumors not involving all three segments or more suitable for hemihepatectomy). Only patients who met the full criteria were included in this LCMM series.

### Perioperative care

All patients underwent comprehensive preoperative laboratory examinations, including complete blood count, biochemical profile, coagulation tests, hepatitis B virus (HBV) and HBV-DNA tests, stool and urine analysis, electrocardiogram (ECG), echocardiography, and chest computed tomography (CT). Preoperative assessment also included Child-Pugh classification and the indocyanine green retention rate at 15 minutes (ICG-R15). Only patients with Child-Pugh grade A or B and ICG-R15 less than 15% were eligible for inclusion in this study.

Liver cirrhosis was defined as liver stiffness measurement (LSM) ≥12.5 kPa on transient elastography, in combination with supporting clinical (e.g., thrombocytopenia), laboratory, and imaging findings (e.g., splenomegaly, nodular liver surface). All patients underwent preoperative LSM assessment.

**Figure 1:**
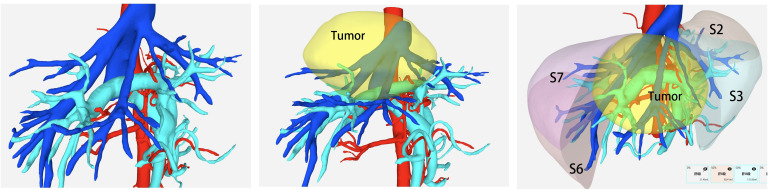
Preoperative 3D reconstruction was utilized to assess critical vascular anatomy and estimate the future liver remnant (FLR) volume.

All patients received contrast-enhanced abdominal CT and magnetic resonance imaging (MRI) preoperatively. In addition, three-dimensional (3D) reconstruction was performed based on enhanced CT images with a slice thickness of 1–2 mm, allowing clear visualization of intrahepatic vascular and biliary structures. Special attention was paid to key vessels, including the main Glissonean pedicles and major hepatic veins (UFV, MHV, and AFV), along with their important branches. Residual liver volume (RLV) and standard liver volume (SLV) were calculated based on 3D reconstruction (**Figure 1**). Surgery was considered only when the future liver remnant (FLR) exceeded 40% of the SLV in patients with chronic liver disease, or cirrhosis, and 30% in patients without liver fibrosis or cirrhosis.

Postoperative management included multimodal analgesia, prophylactic antibiotics (within 24 hours), liver protection, maintenance of internal homeostasis, and nutritional support. Routine nasogastric tube placement was not required. Patients were allowed to drink water once fully awake from anesthesia and were started on a liquid diet on postoperative day 1. Routine laboratory tests, including complete blood count, liver and renal function tests, were performed on postoperative days (POD) 2, 5, and 7 in accordance with institutional protocol to monitor dynamic changes in liver function and detect potential postoperative complications. Drainage fluid bilirubin levels were also monitored regularly to assess for bile leakage. Abdominal ultrasound was routinely performed before the removal of the abdominal drainage tube. Patients were considered fit for discharge once liver function had recovered, no residual infection was present, normal oral intake and bowel function were restored, and adequate ambulation was achieved. After discharge, all patients were followed up at the outpatient clinic every three months. Follow-up evaluations included liver function tests, abdominal ultrasonography, serum alpha-fetoprotein (AFP) levels, and hepatitis B virus (HBV) DNA quantification. Additionally, contrast-enhanced abdominal magnetic resonance imaging (MRI) and chest computed tomography (CT) were performed every six months.

### Operative procedures

The infusion rate was maintained below 75 mL/h from anesthesia induction until the initiation of liver parenchymal transection. During parenchymal transection, central venous pressure (CVP) was controlled between 2 and 4 cmH_2_O (1 cmH_2_O=0.098 kPa). If necessary, intravenous nitroglycerin (0.5–2.0 mg per dose) was administered slowly to assist with CVP reduction while maintaining systolic blood pressure (SBP) above 90 mmHg. In cases where SBP dropped below 90 mmHg, anesthetic agents were reduced, fluid infusion was accelerated, and dopamine was administered to preserve renal perfusion and ensure a urine output of ≥25 mL/h. Upon completion of parenchymal transection, rapid fluid resuscitation was initiated to raise CVP above 5 cmH_2_O.

**Figure 2:**
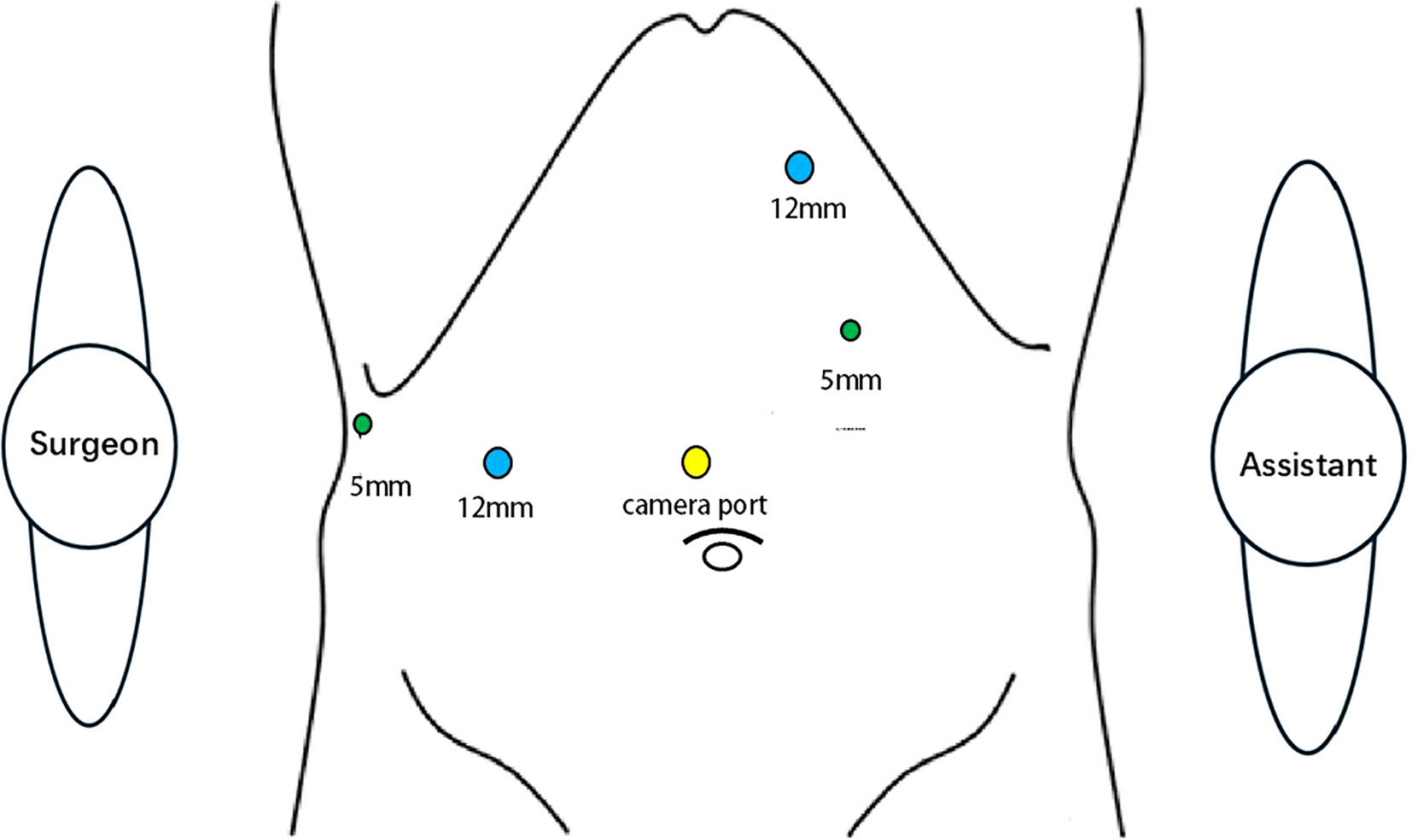
Surgical team positioning and trocar placement during laparoscopic counterclockwise modular mesohepatectomy were standardized. The surgeon stood on the patient’s right side, the first assistant on the left, and the camera operator between the patient’s legs. Trocar placement was adjusted according to the patient’s body habitus and intraoperative requirements.

Following general anesthesia, patients were positioned supine with adjustments to the reverse Trendelenburg position as needed. Pneumoperitoneum was established via a 1-cm supra-umbilical incision, maintaining an intra-abdominal pressure of 14 mmHg. Trocar placement was tailored based on patient body habitus. The surgeon stood on the patient’s right side, the assistant on the left, and the camera operator positioned between the legs (**Figure 2**).

Upon entering the abdominal cavity, adhesions were carefully lysed. The round ligament and falciform ligament were divided to expose the roots of the three major hepatic veins at the second hepatic hilum. Intraoperative laparoscopic ultrasonography was routinely performed to evaluate the relationship between the tumor and major vascular structures and to exclude intrahepatic metastases. The gallbladder was removed, and a hepatic inflow occlusion loop was placed at the first hepatic hilum. The Pringle maneuver was applied intermittently in cycles of 15 minutes of clamping followed by 5 minutes of reperfusion.

After hepatic inflow occlusion, meticulous blunt dissection along the hepatic hilum was performed from right to left using Maryland forceps, following Laennec’s capsule theory. Energy devices were deliberately avoided at this stage to preserve the integrity of Laennec’s capsule and prevent inadvertent entry into the liver parenchyma or the Glissonean sheath. Under laparoscopic magnification, dissection proceeded smoothly within the loose connective tissue plane beneath the capsule. Vascular clips or Hem-o-lok devices were not applied at this stage to avoid interference with subsequent procedures. Short portal vein branches were divided using a Harmonic scalpel (Ethicon, Johnson & Johnson, USA), while small bile ducts were carefully ligated.

To achieve adequate exposure of the right Anterior Glissonean pedicle (AP), the branch to segment 5 (S5 branch) arising from the right posterior pedicle (PPa) was first divided in most cases to expand the operative field. Following isolation, the AP was transected. In cases with limited working space, temporary clamping of the AP with a bipolar clamp was employed to create ischemic demarcation between the right anterior and posterior sectors, which was subsequently marked on the liver surface using electrocautery.

Left-sided liver parenchymal transection was initiated along the falciform ligament using a Harmonic scalpel in combination with bipolar cautery. After dividing the segment 4 branches from the left Glissonean pedicle, the umbilical fissure vein (UFV) was exposed. Dissection was then carefully advanced along the anterior and right lateral walls of the UFV toward the secondary hepatic hilum to expose the root of the middle hepatic vein (MHV), which was transected using an endoscopic linear stapler.

The surgeon then repositioned to the patient’s left side. Dissection continued from the MHV stump toward the right to expose the root of the right hepatic vein (RHV). Upon identification of the RHV, liver parenchymal transection proceeded carefully along the RHV from cranial to caudal direction. Notably, the dorsal branch of the right anterior Glissonean pedicle (P8c) often crosses over the RHV and supplies part of segment 7, complicating the demarcation between segments 7 and 8. Repeated verification using ischemic demarcation or fluorescence counterstaining was therefore necessary to ensure the accuracy of the resection plane. The boundary between segments 5 and 6 was determined by tracing the RHV from the main trunk to its peripheral branches and finally to the liver surface.

**Figure 3:**
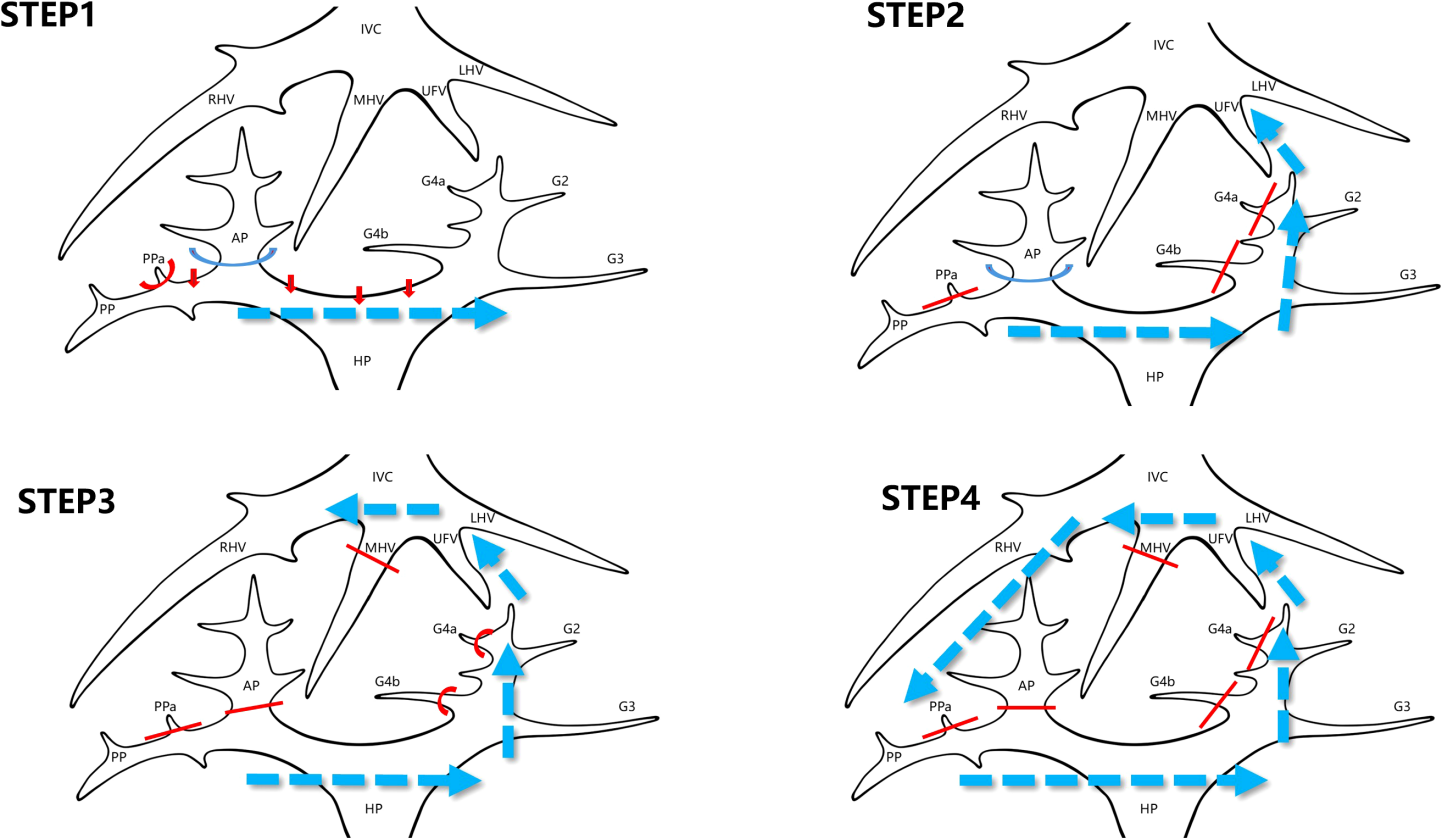
Stepwise surgical sequence of laparoscopic counterclockwise modular mesohepatectomy (LCMM). STEP 1: Dissection of the first hepatic hilum begins along Laennec’s capsule to isolate the right anterior Glissonean pedicle. This step enables identification of the ischemic demarcation line between the right anterior and posterior sectors. STEP 2: Parenchymal transection is initiated from the caudal side along the falciform ligament approach. During this step, Glissonean branches to segment 4 (G4) are divided, and the umbilical fissure vein (UFV) is identified and followed cranially. STEP 3: The middle hepatic vein (MHV) is exposed by tracing the UFV. The right anterior Glissonean pedicle and the root of the MHV are divided using a stapler. Transection continues toward the right side. STEP 4: The surgeon repositions to the patient’s left side. Dissection proceeds along the right hepatic vein (RHV) from cranial to caudal and ventral to dorsal to complete the posterior parenchymal transection. IVC, inferior vena cava; LHV, left hepatic vein; MHV, middle hepatic vein; RHV, right hepatic vein; UFV, umbilical fissure vein; G4, Glissonean pedicle branches to segment 4; AP, anterior Glissonean pedicle; PP, posterior Glissonean pedicle; PPa, Segment 5 branch arising from the right posterior Glissonean pedicle; HP, hepatic pedicle.

**Figure 4:**
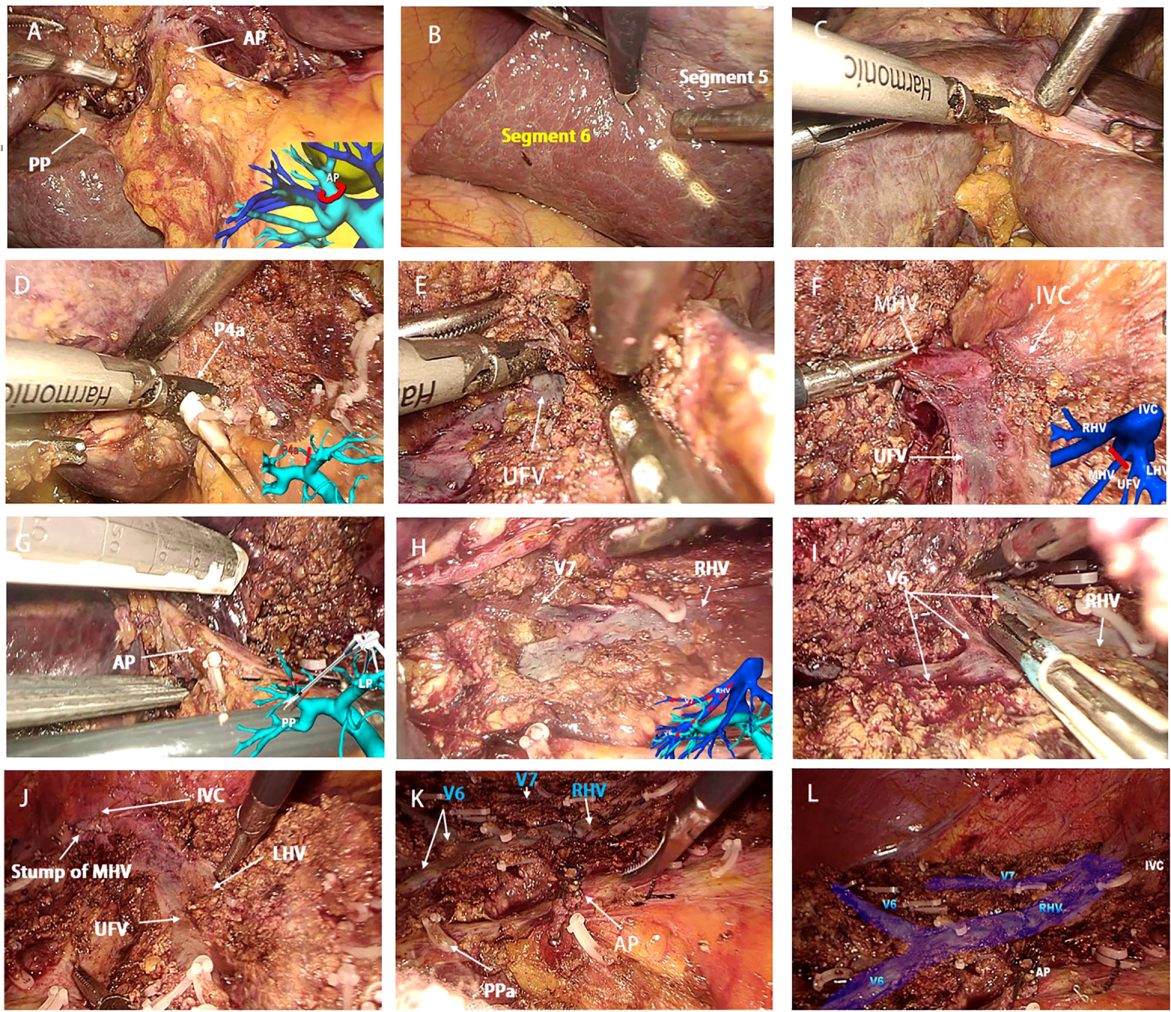
Key intraoperative views of laparoscopic counterclockwise modular mesohepatectomy. **(A, B)** (Step 1): Dissection of the first hepatic hilum along Laennec’s capsule to expose the right anterior Glissonean pedicle and define the ischemic demarcation between the anterior and posterior sectors. **(C–E)** (Step 2): Left-sided parenchymal transection begins along the falciform ligament. Glissonean branches to segment 4 (G4) are divided, and the umbilical fissure vein (UFV) is exposed and followed cranially. **(F, G)** (Step 3): The UFV is traced toward the second hepatic hilum. The root of the middle hepatic vein (MHV) and the right anterior Glissonean pedicle are identified and divided with a stapler. **(H–L)** (Step 4): The surgeon repositions to the patient’s left side. Dissection proceeds along the right hepatic vein (RHV) from cranial to caudal and ventral to dorsal, completing parenchymal transection and specimen removal. UFV, umbilical fissure vein; MHV, middle hepatic vein; RHV, right hepatic vein; V6, segment 6 hepatic vein branch; AP, anterior Glissonean pedicle; PP, posterior Glissonean pedicle; PPa, Segment 5 branch arising from the right posterior Glissonean pedicle.

The laparoscopic counterclockwise modular mesohepatectomy, encompassing segments 4, 5, and 8, was thus successfully completed (**Figures 3**, **4**). A detailed surgical video was provided to further illustrate the procedure.

### Definitions and outcomes

Tumor staging was determined according to the 2023 Chinese guidelines for the diagnosis and treatment of primary liver cancer (16). In addition, pathological T staging was classified based on the 8th edition of the American Joint Committee on Cancer (AJCC) TNM staging system for hepatocellular carcinoma, according to tumor number, tumor size, and the presence or absence of vascular invasion as confirmed in postoperative pathology reports (17). Bile leakage was defined as a total bilirubin concentration in the drainage fluid at least three times higher than the serum total bilirubin concentration on or after postoperative day 3 (18). Posthepatectomy liver failure (PHLF) was defined according to the criteria of the International Study Group of Liver Surgery (ISGLS), as an increase in both international normalized ratio (INR) and serum bilirubin levels on or after postoperative day 5, after excluding other causes of liver dysfunction. severity was classified into three grades based on clinical impact: Grade A required no change in management, Grade B required non-invasive or pharmacological treatment, and Grade C required invasive intervention or life support (19). Exposure of the right hepatic vein (RHV) was defined as complete visualization of the entire main trunk of the RHV throughout its course during surgery. Postoperative complications were classified according to the Clavien–Dindo classification system, which grades complications based on the type and severity of intervention required for management (20).

### Statistics

Continuous variables were expressed as mean ± standard deviation (SD) and compared between groups using the Mann–Whitney U test due to the small sample size. Categorical variables were analyzed using Fisher’s exact test. For key continuous perioperative outcomes, 95% confidence intervals (CIs) were calculated. Survival analysis was performed using the Kaplan–Meier method. A p-value < 0.05 was considered statistically significant. All statistical analyses were conducted using SPSS software version 24.0 (IBM Corp., Armonk, NY, USA) and Python (version 3.11) for confidence interval calculations and graphical presentation.

## Results

**Figure 5:**
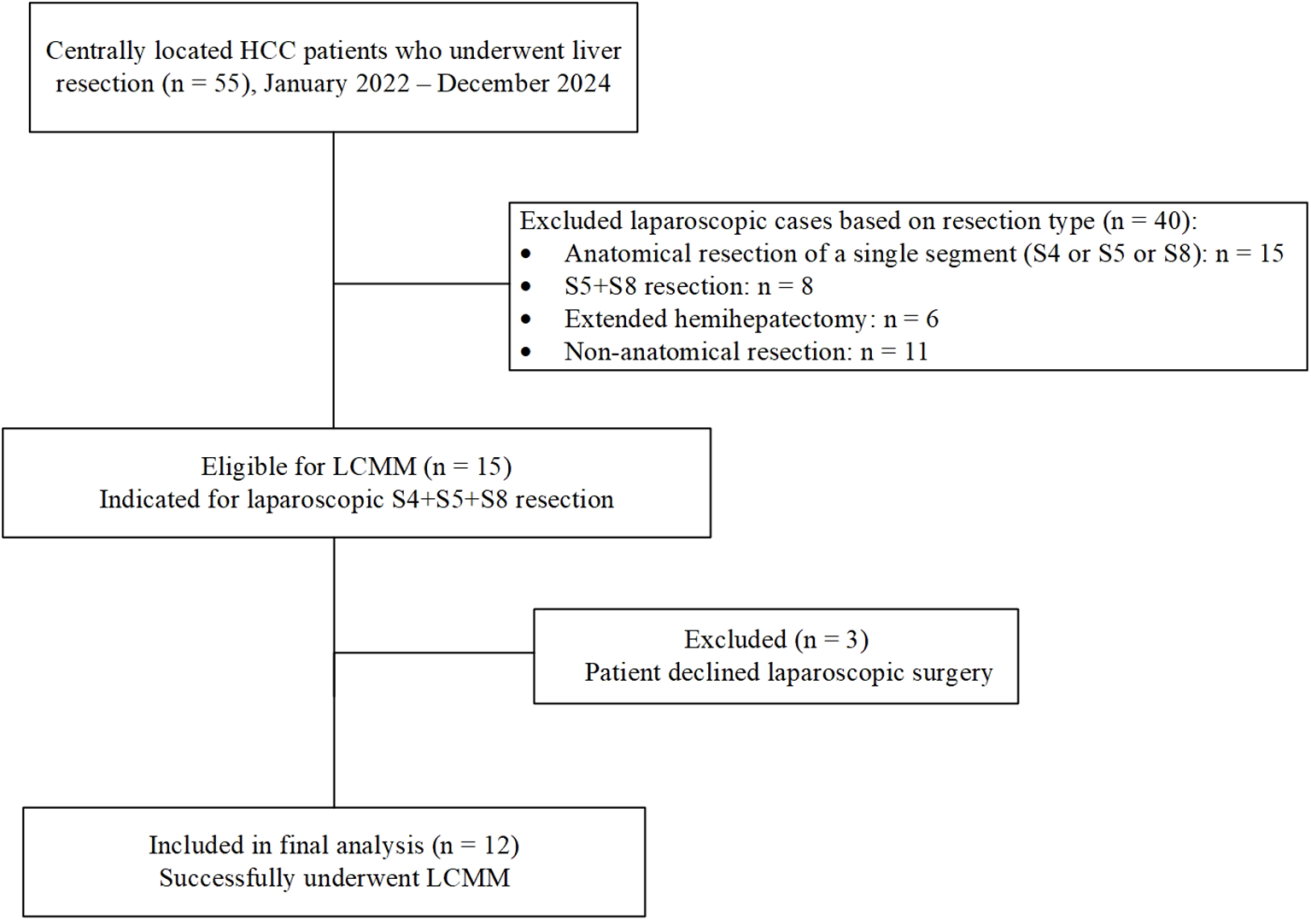
Flowchart of patient selection for laparoscopic counterclockwise modular mesohepatectomy (LCMM). The surgical procedure is demonstrated in the following video link: https://youtu.be/a1u128iMJhk.

Between January 2022 and December 2024, a total of 55 consecutive patients with centrally located hepatocellular carcinoma (HCC) underwent liver resection at our institution. Among them, 15 patients met the eligibility criteria for laparoscopic anatomical resection of segments 4, 5, and 8, and were considered suitable candidates for laparoscopic counterclockwise modular mesohepatectomy (LCMM). However, 3 patients declined laparoscopic surgery. Consequently, 12 patients successfully underwent LCMM and were included in the final analysis. The detailed patient selection process is illustrated in **Figure 5**. The mean age was 62.50 ± 9.09 years, and there were 8 males and 4 females. The mean body mass index (BMI) was 23.16 ± 3.07 kg/m². Detailed patient characteristics are summarized in **Tables 1**, **2**.

**Table 1:** Individual patient characteristics and perioperative outcomes.

Case	Age	Sex (M/F)	HBsAg	Liver cirrhosis	ICG R15 (%)	LSM(Kpa)	Previous treatment	Tumor number	Tumor size (cm)	AJCC8 T Stage	CNLC	FLR(%)	Pathological MVI	Ishak Fibrosis Score	Operative time (min)	Blood loss (ml)	Pringle maneuver duration (min)	Postoperative complications	POS (days)
1	43	M	+	+	8.9	15.7	–	1	6.9	T1b	Ib	52.5	–	F6	259	155	65	–	7
2	72	M	+	–	5.6	14.4	–	3	7.4	T3	IIa	58.2	+	F4	287	210	65	–	9
3	68	M	–	+	4.5	7.7	–	1	5.8	T1b	Ib	49.6	–	F2	288	355	88	–	6
4	55	F	+	+	6.7	16.7	TACE	4	4.7	T2	IIb	57.7	+	F6	195	30	33	Bile leakage	21
5	58	M	+	–	1.8	12.8	–	1	5.6	T1b	Ib	54.1	–	F4	168	80	45	–	11
6	67	M	+	+	11.3	18.8	–	2	7.2	T3	IIa	47.3	–	F6	313	290	89	Liver failure	10
7	71	F	–	+	3.9	10.5	–	1	4.7	T1b	Ib	43.8	–	F3	187	120	40	–	5
8	75	M	+	–	5.7	12.9	–	2	8.8	T3	IIa	44.7	–	F4	334	340	78	–	13
9	55	M	–	–	2.1	8.3	–	1	7.0	T1b	Ib	45.3	–	F2	267	270	77	–	7
10*	61	F	+	+	9.4	17.3	TACE	4	5.5	T3	IIb	57.7	+	F6	284	135	63	Pneumonia	12
11	59	M	–	+	4.4	9.2	–	1	6.3	T1b	Ib	55.6	–	F4	249	45	60	–	9
12	66	F	+	+	3.4	15.4	–	1	5.3	T1b	Ib	60.1	–	F6	213	105	45	–	8

HBsAg, Hepatitis B surface antigen; ICG-R15,Indocyanine green retention test after 15 min; LSM, Liver Stiffness Measurement; TACE transarterial chemoembolization; T staging was classified according to the 8th edition of the AJCC TNM staging system for hepatocellular carcinoma; CNLC, China Liver Cancer Staging; FLR, Future liver remnant; MVI, microscopic vascular invasion; POS postoperative stays.

*Case No. 10: Three central tumors resected; one lesion in segment 6 treated with intraoperative microwave ablation (MWA).

**Table 2:** Patient demographics and clinical characteristics.

Variables	Number	95%CIs
Patients	12	
Age (years)	62.50±9.09	56.72- 68.28
Sex (M/F)	8/4	
ASA		
II	5	
III	7	
BMI (kg/m^2^)	23.16±3.07	21.21- 25.12
Diabetes	3	
Smoking	4	
Alcohol abuse	2	
HBsAg (+)	8	
Liver cirrhosis	8	
Child’s Grading A	12	
ICG R15(%)	5.64±2.95	3.76- 7.52
LSM (Kpa)	13.30±3.70	10.96- 15.66
Abdominal surgery history	3	
Previous treatment	2	
FLR (%)	52.21±5.87	48.49- 55.95
Ishak Fibrosis Score		
F0~5	7	
F6	5	
AJCC8 T Stage		
T1b	7	
T2	1	
T3	4	
CNLC Tumor staging		
CNLC Ib	7	
CNLC IIa	3	
CNLC IIb	2	
Tumor size (cm)	6.26±1.22	5.49- 7.05
Pathological MVI	3	

ASA, American society of anesthesiologists; BMI, Body mass index; HBsAg, Hepatitis B surface antigen; ICG-R15, Indocyanine green retention test after 15 min; LSM, Liver Stiffness Measurement; FLR, Future liver remnant; T staging was classified according to the 8th edition of the AJCC TNM staging system for hepatocellular carcinoma; CNLC, China Liver Cancer Staging; MVI, microscopic vascular invasion.

Comorbidities included diabetes mellitus in three patients, all of whom achieved effective perioperative glycemic control with insulin. Eight patients were hepatitis B surface antigen (HBsAg) positive, two had a history of long-term alcohol consumption, and eight patients were clinically diagnosed with liver cirrhosis based on liver stiffness measurement and imaging findings, among whom five were confirmed with Ishak stage F6 fibrosis on postoperative pathology. All patients had preserved liver function classified as Child–Pugh A, with a mean indocyanine green retention rate at 15 minutes (ICG R15) of 5.64 ± 2.95%. Three patients had a history of laparoscopic cholecystectomy, and two patients with tumor stage IIb received transcatheter arterial chemoembolization (TACE) prior to surgery. The mean tumor diameter was 6.26 ± 1.22 cm.

All procedures were completed laparoscopically without conversion to open surgery or intraoperative blood transfusion. Among the patients with multifocal tumors, Case 4 had four lesions, all located within the central sector (segments 4, 5, and 8), and complete resection was achieved through laparoscopic mesohepatectomy. In Case 10, three of the four tumors were located centrally and were resected together with the main specimen; the fourth lesion, located in segment 6, was treated with intraoperative microwave ablation (MWA). Postoperative imaging confirmed complete ablation, and pathological assessment confirmed R0 resection in all patients.

The mean operative time was 253.66 ± 52.47 minutes, with a mean Pringle maneuver duration of 62.33 ± 18.61 minutes. The mean intraoperative blood loss was 177.91 ± 112.76 mL. The right hepatic vein (RHV) was successfully exposed in all patients, and the umbilical fissure vein (UFV) was exposed in 8 cases.

The average postoperative hospital stay was 9.83 ± 4.26 days. Three patients experienced postoperative complications: one case of posthepatectomy liver failure (PHLF), classified as ISGLS Grade B, which was managed with hepatoprotective agents; one case of pneumonia on postoperative day 4 that resolved with antibiotic therapy; and one case of bile leakage on postoperative day 5. The bile leakage was treated with ultrasound-guided percutaneous drainage under local anesthesia, fulfilling the criteria for Clavien–Dindo Grade IIIa. The drainage tube was removed two months later in an outpatient setting. No postoperative mortality occurred within 30 or 90 days. Detailed outcomes are summarized in **Table 3**.

**Table 3:** Intraoperative and postoperative outcomes.

Variables	Number	95%CIs
Operative time (min)	253.66±52.47	220.33- 287.01
Pringle maneuver duration (min)	62.33±18.61	50.51- 74.16
Blood loss (mL)	177.91±112.76	106.27- 249.56
Exposure of right hepatic vein	12	
Exposure of umbilical fissure vein	8	
Surgical margin R0	12	
Hospital stay(days)	9.83±4.26	7.13- 12.54
Postoperative complications	3	
Clavien–Dindo Classification		
Grade I	0	
Grade II	2	
Liver failure	1	
Pneumonia	1	
^†^Grade IIIa	1	
Bile leakage	1	
Grade IIIb-V	0	

^†^Bile leakage case was classified as Clavien–Dindo IIIa and managed with ultrasound-guided percutaneous drainage under local anesthesia.

An exploratory comparison of clinical and intraoperative variables between patients with and without postoperative complications is presented in **Table 4**. Patients who developed complications had significantly higher liver stiffness measurement (LSM) values (17.60 ± 1.08 vs. 11.88 ± 3.05 kPa, p = 0.009), higher indocyanine green retention at 15 minutes (ICG-R15) (9.13 ± 2.31% vs. 4.48 ± 2.14%, p = 0.018), and were more likely to have Ishak stage F6 fibrosis (p = 0.045). Other variables, including age, BMI, FLR, tumor size, operative time, Pringle maneuver duration, blood loss, and abdominal surgery history, did not differ significantly between groups.

**Table 4:** Exploratory comparison of clinical variables in patients with and without postoperative complications.

Variables	With complications(n=3)	Without complications(n=9)	P
Age (years)	61.00±6.00	63.00±10.17	0.643
BMI (kg/m^2^)	25.60±3.14	22.36±2.75	0.138
Abdominal surgery history	1	2	1.000
Ishak F6	3	2	0.045^*^
ICG R15 (%)	9.13±2.31	4.48±2.14	0.018^*^
LSM (Kpa)	17.60±1.08	11.88±3.05	0.009^*^
FLR (%)	54.23±6.00	51.54±6.03	0.578
Tumor size (cm)	5.80±1.28	6.42±1.25	0.517
Operative time (min)	264.00±61.49	250.22±52.80	0.727
Pringle maneuver duration (min)	61.67±28.02	62.56±16.73	1.000
Blood loss (mL)	151.67±130.80	186.67±113.41	0.727

*p < 0.05, statistically significant.

The follow-up period ranged from 6 to 23 months. During this period, no patient died, and all were alive at the last follow-up. Kaplan–Meier analysis estimated a mean disease-free survival (DFS) of 18.75 months (95% CI: 15.41–22.10 months); However, the median DFS was not reached. Tumor recurrence occurred in four patients. Among them, one developed pulmonary metastasis 8 months postoperatively and received combination targeted therapy and immunotherapy. Another experienced an isolated intrahepatic recurrence in the right liver at 11 months, managed with percutaneous ablation under ultrasound guidance, followed by two sessions of TACE and long-term combination therapy. The remaining two patients developed multiple intrahepatic metastases at 13 and 17 months, respectively, and underwent TACE combined with targeted and immunotherapy.

## Discussion

Over recent decades, ongoing advances in surgical techniques and evolving paradigms in hepatobiliary surgery have led to the increasing recognition and adoption of central hepatectomy (CH) in clinical practice. The primary objective of CH is to preserve as much functional liver parenchyma as possible while achieving oncological clearance, thereby reducing the risk of posthepatectomy liver failure (21). Multiple studies have demonstrated that CH is associated with a significantly lower incidence of PHLF and shorter hospital stays compared to conventional major hepatectomy, contributing to faster postoperative recovery (14, 21, 22). Despite these benefits, CH remains technically challenging, often requiring longer operative times and associated with a higher incidence of bile leakage, largely due to the complex central hepatic anatomy (14, 23). Nevertheless, existing evidence suggests that CH achieves comparable perioperative mortality rates and long-term oncologic outcomes relative to standard liver resection (22).

Among various CH procedures, resection of Couinaud segments 4, 5, and 8 also known as central bisectionectomy or central zone II resection—is considered one of the most challenging anatomical liver resections (24). This procedure involves a wide transection plane extending along both the left and right hepatic veins (LHV and RHV), traversing the central part of the liver where vascular and biliary structures are densely concentrated. The limited operative field and the proximity to major vascular structures, particularly the middle hepatic vein (MHV) and RHV, make central bisectionectomy technically complex.

Laparoscopic surgery is minimally invasive, characterized by smaller incisions, reduced postoperative pain, and attenuated surgical stress responses. These advantages contribute to faster postoperative recovery and shorter hospital stays, aligning well with the principles of Enhanced Recovery After Surgery (ERAS) (25, 26). As a result, laparoscopic approaches have been increasingly adopted in clinical practice. In this study, we retrospectively analyzed our initial experience and clinical outcomes of LCMM for HCC located in the central liver.

Our results demonstrated that the LCMM technique is both safe and feasible. It enables precise anatomical resection of complex central liver segments while preserving anatomical integrity and ensuring oncological radicality. Moreover, patients experienced favorable postoperative recovery with satisfactory short-term outcomes.

In an exploratory subgroup analysis, we compared clinical variables between patients with and without postoperative complications (**Table 4**). The findings suggested that higher liver stiffness measurement (LSM), elevated ICG-R15 values, and advanced liver fibrosis (Ishak stage F6) may be associated with a greater risk of postoperative complications. Although these trends are clinically plausible, the limited sample size reduces the statistical power and restricts generalizability. These observations should therefore be interpreted with caution and considered hypothesis-generating, pending validation in larger prospective studies.

In 1802, French surgeon Laennec first described Laennec’s capsule as an intrinsic membrane of the liver, distinct from the serosa, which envelops the entire liver surface. However, for a long time, Laennec’s capsule was misinterpreted as either the serosa or the Glissonean sheath. In 2017, Sugioka et al. further confirmed that Laennec’s capsule not only covers the liver surface but also extends to form potential spaces around the hepatic plate, Glissonean pedicles, hepatic veins, and the inferior vena cava (IVC). They proposed that Laennec’s capsule could serve as a critical anatomical landmark for the isolation and exposure of Glissonean pedicles during liver surgery (27, 28). However, in patients with cirrhosis or distorted hepatic anatomy, identification of Laennec’s capsule may be more challenging due to fibrotic changes and tissue adhesions. In such cases, careful blunt dissection under laparoscopic magnification, repeated anatomical verification, and adjunctive techniques such as intraoperative ultrasonography or indocyanine green (ICG) fluorescence imaging may aid in accurate identification and help minimize the risk of misdissection.

The Glissonean approach is a fundamental technique in anatomical liver resection (29). Compared with the traditional intrafascial dissection, it offers notable advantages, including reduced intraoperative blood loss and shorter liver transection time (30). In conventional Glissonean approaches, partial liver parenchymal dissection is often required to expose the targeted Glissonean pedicle. However, with the application of Laennec’s capsule theory, the Glissonean pedicle can be fully isolated within the potential space of Laennec’s capsule in most cases, without the need for parenchymal transection. This approach significantly reduces the risk of injury to the bile ducts and vessels within the Glissonean sheath, thereby improving the safety, precision, and standardization of anatomical liver resection (31).

In the LCMM procedure, the first and critical step is to perform blunt dissection under Pringle maneuver occlusion to precisely enter the Laennec’s membrane plane between the liver parenchymal surface and the Glissonean pedicle. This technique allows for rapid and effective control of the right anterior Glissonean pedicle, enabling clear identification of the ischemic demarcation between the right anterior and right posterior sections, thus providing an anatomical basis for accurate parenchymal transection. Under the magnified view of laparoscopy, this step is simple, reproducible, and easy to master, typically completed within 3–5 minutes. It is recommended to perform blunt dissection throughout the procedure to minimize the risk of inadvertent injury. Without hepatic inflow occlusion, bleeding from the dissection surface may occur, impairing visualization and increasing the risk of entering the wrong anatomical plane or inadvertently dissecting into the Glissonean pedicle, thereby compromising surgical safety and outcomes. Additionally, placing the occlusion tape around the hepatic pedicle provides downward traction on the hepatoduodenal ligament, which facilitates entry into the Laennec’s membrane plane and further enhances the safety and operability of the procedure.

The hepatic veins serve as essential anatomical boundaries for Couinaud’s liver segmentation and are crucial landmarks in anatomical liver resection (ALR). Precise exposure of major hepatic veins is a key step to ensure the safety and accuracy of ALR (13). During mesohepatectomy, exposing the right hepatic vein (RHV) is often one of the most challenging parts of the procedure. Based on our clinical experience, exposure of the RHV is associated with a higher risk of bleeding compared to the middle hepatic vein (MHV). This is mainly attributed to the anatomical characteristics of the RHV, which drains directly into the inferior vena cava (IVC) and is typically located inferior to the IVC. Inadequate control of central venous pressure (CVP) during surgery may easily result in blood reflux from the IVC, leading to massive bleeding. Additionally, most branches of the RHV run ventrally, making them prone to tearing during dissection or traction, further increasing the risk of hemorrhage. Typically, both transection planes during mesohepatectomy are created from the caudal to cranial direction. In this study, the left transection plane was approached through the falciform ligament and dissected in a caudal-to-cranial direction. After dividing the Glissonean pedicles of segment 4, the dissection was continued along the umbilical fissure vein (UFV), providing direct and rapid access to the root of the middle hepatic vein (MHV). Subsequently, dissection was continued toward the right side until the root of the right hepatic vein (RHV) was fully exposed. The parenchymal transection was then performed along the RHV from cranial to caudal, allowing continuous exposure of the vein during liver resection. This approach enables immediate and clear visualization of the RHV, allowing the vein to serve as a critical anatomical guide during deep parenchymal transection. Compared with the conventional caudal approach, this technique significantly reduces the risk of RHV injury and hemorrhage caused by excessive traction on the liver parenchyma (32). In this study, the right hepatic vein (RHV) was fully exposed in all cases (100%), with no major hemorrhagic events observed. In our cohort, the operative time was shorter and intraoperative blood loss was less than previously reported in the literature. Notably, no patients required blood transfusion or conversion to open surgery, demonstrating the safety and efficiency of this surgical approach (33, 34).Notably, no patients required blood transfusion or conversion to open surgery, demonstrating the safety and efficiency of this surgical approach (33, 34).

In terms of postoperative complications, the overall morbidity rate in our series was 25%, with only one major complication (Clavien-Dindo grade IIIa). One patient developed bile leakage and was successfully managed with ultrasound-guided percutaneous drainage. The leakage site could not be confirmed without ERCP. This patient had multiple risk factors, including liver cirrhosis (LSM 16.7 kPa, Ishak F6), advanced tumor stage (T2), and prior TACE. Another patient (Case No. 6) experienced ISGLS Grade B posthepatectomy liver failure (PHLF) despite a preoperative ICGR-15 of 11.3%. The relatively small FLR (47.3%), cirrhosis (LSM 18.8 kPa), prolonged operative and ischemia time, and previous TACE may have contributed. Both patients recovered fully with conservative treatment. A third patient developed a pulmonary infection, likely related to smoking history, and recovered with antibiotics.

These outcomes are comparable or favorable to those reported in similar studies. Masuda et al (35). reported a bile leakage rate of 33% in laparoscopic and 42% in open central hepatectomy. For pulmonary complications, Wang et al (36). found an incidence of up to 27%. In contrast, our rates of bile leakage and pulmonary infection (each 8.3%) are relatively low, highlighting the safety of the LCMM technique in carefully selected patients.

Recent studies have also supported the oncological feasibility and safety of laparoscopic anatomical mesohepatectomy (LAMH), which shares similar principles with our LCMM approach. Li et al. (11) conducted a propensity score-matched study comparing laparoscopic versus open mesohepatectomy in patients with centrally located hepatocellular carcinoma (HCC). Their results demonstrated that the laparoscopic approach achieved comparable long-term outcomes, including disease-free survival and overall survival, while offering the advantages of reduced intraoperative blood loss and faster postoperative recovery. In a more recent study, Siow et al. (33) confirmed the feasibility and safety of laparoscopic central hepatectomy in selected patients. Despite the technical complexity, the procedure was associated with low perioperative morbidity and satisfactory short-term oncological outcomes. According to our findings, most patients were classified as stage Ib. The follow-up period ranged from 6 to 23 months. Kaplan–Meier analysis estimated a mean disease-free survival (DFS) of 18.75 months (95% CI: 15.41–22.10 months); the median DFS was not reached. Despite the limited sample size and short-to-moderate follow-up, the long-term oncological outcomes of the LCMM procedure were favorable, with only four patients experiencing recurrence. These findings are consistent with previously published literature and further support the oncological feasibility of LCMM for centrally located HCC in high-volume, experienced centers.

Compared with traditional laparoscopic middle hepatectomy, LCMM demonstrates several notable advantages. Traditional laparoscopic techniques typically rely on direct intrahepatic dissection, which can make early identification and control of hepatic pedicle vessels technically challenging. In contrast, LCMM employs an extraglissonian approach via Laennec’s capsule, enabling early and clear exposure of the right anterior hepatic pedicle. This facilitates early vascular control, allowing precise delineation of ischemic demarcation lines and improving surgical planning while minimizing the risk of intraoperative vascular injury. Furthermore, early ligation of major portal vein branches interrupts tumor blood supply at the outset of the procedure, which may reduce the risk of intrahepatic tumor dissemination—a potential concern with conventional laparoscopic techniques that often achieve vascular control at a later stage. Anatomically, LCMM takes advantage of the fixed position and minimal variation of segment 4 (G4) as a natural transection plane on the left side. By initiating parenchymal transection along the umbilical fissure vein (UFV), the middle hepatic vein (MHV) can be precisely identified and isolated, reducing the risk of repeated dissection or injury due to uncertain anatomical orientation.

After division of the MHV, the root of the right hepatic vein is easily visualized, and a cranial approach allows for controlled and safe exposure of hepatic venous structures. This strategy minimizes the risk of hepatic vein avulsion, which is among the most dangerous complications associated with laparoscopic liver resections. In our series, no conversions to open surgery were required, and intraoperative blood loss was minimal, indicating the safety and technical stability of LCMM. Collectively, these findings highlight the procedural advantages of LCMM in terms of anatomical clarity, oncological control, and perioperative outcomes. The modular counterclockwise design, combined with extraglissonian entry and early structural separation, offers a systematic and reproducible approach that addresses key limitations of traditional laparoscopic techniques.

This study has several limitations that should be acknowledged. First, it was a single-arm, single-center retrospective study without a comparison group, such as open surgery or other laparoscopic approaches. The absence of a control group limits the ability to directly compare the outcomes of LCMM with other surgical techniques and thus affects the generalizability of the findings. Second, the overall sample size was relatively small. However, it should be noted that anatomical laparoscopic resection of segments 4, 5, and 8, as performed in LCMM, is technically demanding and relatively uncommon, even in high-volume centers. The rarity of this procedure reflects the complexity of centrally located tumors and the challenges associated with precise dissection near major hepatic vessels. Despite the limited number of cases, this series provides meaningful preliminary evidence regarding the feasibility, safety, and short-term oncological efficacy of the LCMM technique. Finally, the follow-up duration was relatively short, with a maximum of 23 months. Nevertheless, all patients remain under active follow-up, and long-term oncological outcomes, including overall survival (OS) and disease-free survival (DFS), are being prospectively collected for future analysis.

All procedures in this study were performed by a single highly experienced hepatobiliary surgeon specializing in laparoscopic anatomical liver resection. While this ensured technical consistency and minimized operator-related variability, it also raises questions regarding the learning curve and reproducibility of the LCMM technique in broader clinical practice. Given the technical complexity of laparoscopic mesohepatectomy, especially for central tumors, we acknowledge that a significant learning curve is likely required. Based on our institutional experience, approximately 5–10 cases may be needed for experienced laparoscopic liver surgeons to become proficient with the LCMM technique. The modular design and standardized dissection sequence of LCMM, guided by clear anatomical landmarks such as Laennec’s capsule and major hepatic veins, may help facilitate training and adoption among qualified surgeons. Future studies involving multiple surgeons and institutions are warranted to assess inter-operator reproducibility and to further validate the learning curve for safe implementation. In addition, potential technical pitfalls should be acknowledged, including the risk of bleeding near major hepatic veins and the potential for bile duct injury during Glissonean pedicle dissection. Careful anatomical planning and stepwise execution are essential to mitigate these risks and ensure safe adoption in various surgical settings.

In conclusion, laparoscopic counterclockwise modular mesohepatectomy (LCMM) appears to be a safe, feasible, and standardized surgical technique for centrally located hepatocellular carcinoma (HCC). By optimizing the dissection sequence and incorporating Laennec’s capsule theory under laparoscopic magnification, this approach enables precise vascular control and minimizes intraoperative risks. Our preliminary findings support its use in experienced centers; however, further large-scale, controlled studies are necessary to validate its long-term oncological benefits and broader applicability. Moreover, due to its modular anatomical design and early vascular control, LCMM may be suitable as a conversion approach for initially unresectable centrally located HCC. Its compatibility with preoperative downstaging strategies (e.g., TACE or systemic therapy) also supports its potential integration into neoadjuvant protocols, although further prospective validation is warranted.

## Data Availability

The original contributions presented in the study are included in the article/supplementary material. Further inquiries can be directed to the corresponding author.
